# Normal development of mice lacking PAXX, the paralogue of XRCC4 and XLF

**DOI:** 10.1002/2211-5463.12381

**Published:** 2018-02-04

**Authors:** Raquel Gago‐Fuentes, Mengtan Xing, Siri Sæterstad, Antonio Sarno, Alisa Dewan, Carole Beck, Stefano Bradamante, Magnar Bjørås, Valentyn Oksenych

**Affiliations:** ^1^ Institute of Clinical and Molecular Medicine (IKOM) Laboratory Center Norwegian University of Science and Technology Trondheim Norway; ^2^ St. Olavs Hospital Clinic of Medicine Trondheim University Hospital Trondheim Norway; ^3^ Department of Microbiology Oslo University Hospital University of Oslo Oslo Norway; ^4^ Present address: Centre for Immune Regulation and Department of Immunology Oslo University Hospital‐Rikshospitalet University of Oslo Oslo Norway; ^5^ Present address: KG Jebsen Coeliac Disease Research Centre University of Oslo Oslo Norway

**Keywords:** C9orf142, Cernunnos, lymphocyte, mouse genetics, T‐FISH, XLS

## Abstract

DNA repair consists of several cellular pathways which recognize and repair damaged DNA. The classical nonhomologous DNA end‐joining (NHEJ) pathway repairs double‐strand breaks in DNA. It is required for maturation of both B and T lymphocytes by supporting V(D)J recombination as well as B‐cell differentiation during class switch recombination (CSR). Inactivation of NHEJ factors Ku70, Ku80, XRCC4, DNA ligase 4, DNA‐PKcs, and Artemis impairs V(D)J recombination and blocks lymphocyte development. Paralogue of XRCC4 and XLF (PAXX) is an accessory NHEJ factor that has a significant impact on the repair of DNA lesions induced by ionizing radiation in human, murine, and chicken cells. However, the role of PAXX during development is poorly understood. To determine the physiological role of PAXX, we deleted part of the *Paxx* promoter and the first two exons in mice. Further, we compared *Paxx*‐knockout mice with wild‐type (WT) and NHEJ‐deficient controls including *Ku80‐* and *Dna‐pkcs*‐null and severe combined immunodeficiency mice. Surprisingly, *Paxx*‐deficient mice were not distinguishable from the WT littermates; they were the same weight and size, fertility status, had normal spleen, thymus, and bone marrow. *Paxx*‐deficient mice had the same number of chromosomal and chromatid breaks as WT mice. Moreover, *Paxx*‐deficient primary B lymphocytes had the same level of CSR as lymphocytes isolated from WT mice. We concluded that PAXX is dispensable for normal mouse development.

**Abbreviations**
53BP1: p53‐binding protein 1
AID: activation‐induced cytidine deaminase
ATM: Ataxia telangiectasia mutated
CSR: class switch recombination
DDR: DNA damage response signaling
DNA‐PKcs: DNA‐dependent protein kinase, catalytic subunit
DNA‐PK: DNA‐dependent protein kinase (holoenzyme)
DSB: DNA double‐strand break
HRP: Horseradish peroxidase
Ig: immunoglobulin
IL: interleukin
Lig4: DNA ligase 4
LPS: lipopolysaccharide
NHEJ: nonhomologous end joining
PAXX: paralogue of XRCC4 and XLF
RAG1/2: recombination activating genes 1 and 2
SCID: severe combined immunodeficiency
T‐FISH: telomere fluorescence *in situ* hybridization
UNG: uracil DNA N‐glycosylase
XLF: XRCC4‐like factor
XLS: XRCC4‐like small protein
XRCC4: X‐ray cross‐complementing protein 4

Nonhomologous end joining (NHEJ) recognizes and repairs DNA double‐strand breaks (DSBs) throughout the cell cycle 1. NHEJ is required to maintain genomic stability in response to extrinsically and physiologically induced DSBs. The latter includes DNA breaks generated by the recombination activating genes (RAG1/2) during V(D)J recombination in developing B and T lymphocytes. Activation‐induced cytidine deaminase (AID) converts cytosine to uracil at the actively transcribed switch regions of immunoglobulin heavy‐chain coding regions in mature B lymphocytes, and in cooperation with uracil DNA N‐glycosylase (UNG), it produces single‐strand breaks in both DNA strands leading to DSBs that are recognized and repaired by NHEJ 1, 2. NHEJ includes core subunits, Ku70 and Ku80 (or X‐ray repair cross‐complementing proteins, XRCC6 and XRCC5, respectively) that form the Ku heterodimer, which recognizes DSBs and serves as a platform to recruit and stabilize other NHEJ subunits. X‐ray cross‐complementing protein 4 (XRCC4) and DNA ligase 4 (Lig4) form another heterodimer that ligates DNA ends. There are several known accessory NHEJ factors that likely work downstream of Ku, upstream or in cooperation with XRCC4/Lig4, and are required in specific cases. Among them DNA‐dependent protein kinase, catalytic subunit (DNA‐PKcs), which is a protein kinase that forms the DNA‐PK holoenzyme with Ku70/Ku80 and phosphorylates most NHEJ factors, including itself. DNA‐PKcs is specifically required for stabilization and activation of the nuclease Artemis that processes RAG‐induced DNA hairpins and overhangs during V(D)J recombination. The inactivation of any core NHEJ factor, as well as DNA‐PKcs and Artemis, results in severe immunodeficiency associated with B and T lymphocytopenia, due to the inability of B and T progenitors to perform V(D)J recombination and thus to mature (reviewed in Ref. 1).

XRCC4‐like factor (XLF, also known as Nhej1 or Cernunnos) is considered both a core and an accessory factor in NHEJ. Similar to core NHEJ factors, XLF is evolutionary conserved in eukaryotic cells from yeast to humans. It also suppresses medulloblastoma development in p53‐deficient background 3. On the other hand, *Xlf* inactivation alone does not lead to a severe phenotype in mice, likely due to its functional overlap with other accessory NHEJ factors 4, 5 and potentially with the Ataxia telangiectasia mutated (ATM)‐dependent DNA damage response (DDR) pathway 1, 6, 7, 8. XLF was also shown to have functional overlap with RAG recombinase, which is likely lymphocyte‐specific 9. *Xlf* inactivation in combination with, for example, knockout of *Atm*, histone *H2ax*, DNA damage response factor *p53‐binding protein 1* (*53BP1*), or RAG2 truncation leads to a block in lymphocyte development and thus a severe reduction in B and T lymphocyte numbers 1, 5, 9. It is very likely that other accessory NHEJ or DDR factors complement the roles of XLF in DNA repair and lymphocyte development.

PAXX (also known as C9Orf142, or XRCC4‐like small protein) is an accessory NHEJ factor reported by several research groups in 2015 10, 11, 12. *PAXX*‐deficient human, murine, and chicken cells displayed an increased sensitivity to DSBs induced by ionizing radiation 10, 11, 12. In experiments based on knockout chicken and murine cells, PAXX was shown to have some functional redundancy with its paralogue XLF 12, 13, 14, 15, 16. In addition, three knockout mouse models were generated at different laboratories and published recently 17, 18, 19. These studies suggest that PAXX has an overlapping function with XLF and is required for embryonic development and maintenance of central nervous system. However, the role of PAXX on the organismal level requires further investigation.

To determine the physiological role of PAXX, we generated a *Paxx*‐deficient knockout mouse model. We compared *Paxx* null mice with wild‐type (WT) and NHEJ‐deficient controls, including the *Ku80*‐, *Dna‐pkcs*‐deficient, and severe combined immunodeficiency (SCID) mice. We found that *Paxx* null mice do not differ from WT and heterozygous littermates in viability, lymphoid organ development, class switch recombination (CSR) efficiency, and genomic stability.

## Materials and methods

### Mouse models

All experiments involving mice were performed according to the protocols approved by the Norwegian University of Science and Technology (NTNU). *Ku80* ^*+/−*^ 20, *Dna‐pkcs* ^*+/−*^ 21, SCID 22, and *Ung* ^*+/−*^ 23 mice were described previously. *Paxx* ^*+/−*^ mice are custom‐generated and described here for the first time.

### Generation of *Paxx* ^*−/−*^ mice

*Paxx*‐deficient (*Paxx* ^*−/−*^) mice were generated upon request as OKS1 project by genOway (Lyon, France). Analysis of the *Paxx* gene structure showed that another gene *Clic3* overlaps with the *Paxx*. Thus, only the part of *Paxx* gene that does not overlap with *Clic3* was deleted. Two sgRNAs were designed to target the promoter region and the end of exon 2 of the *Paxx* gene: sgRNA#1, CCC AAG GGC TTG TAC TGC; sgRNA#2, GGC GGC GTC CGT CAC ACT. Fertilized oocytes were collected from superovulated female mice previously mated with males. The purified sgRNAs and Cas9 RNA were microinjected into the male pronucleus. Injected zygotes were cultivated overnight to the two‐cell stage to assess sgRNAs toxicity. Resulting two‐cell embryos were reimplanted into pseudopregnant foster mothers 0.5 day *post coitum*. A total of 158 injected embryos were reimplanted into foster mothers, leading to the birth of 60 viable pups.

### Mouse screening strategy

The screening was performed on genomic DNA extracted from mouse skin. Two primers were used to amplify the original or modified part of the *Paxx* gene. The intact gene results in a 965‐bp product and deletion resulted in shorter products ranging from 280 to 412 bp, depending on the size of deletion. Four founder mice were identified in which mutation was confirmed by sequencing at genOway. Three heterozygous *Paxx*‐knockout lines were obtained by backcrossing founders to C57BL6/N WT mice. The primers to detect deletion in murine *Paxx* gene were as follows: ACA GAG GGT GGT GAC TCA GAC AAT GG and GGA AAT GCT ATT AGA ACC ACT GCC ACG.

### Antibodies

To detect the PAXX protein by western blot, we used rabbit polyclonal anti‐PAXX/C9orf142 IgG (NovusBio, Littleton, CO, USA, NBP1‐94172, dilution 1 : 500), which recognizes the C‐terminal half of the PAXX protein (amino acids 109‐204); anti‐PAXX/C9orf142 IgG (Abcam, Cambridge, UK, ab126353, 1 : 200) and swine polyclonal anti‐rabbit Ig‐HRP (Dako antibodies, #P0399, 1 : 3000). Anti‐GAPDH rabbit polyclonal (Sigma, St. Louis, MO, USA, #G9545, developed to recognize 314–333 amino acids of mouse GAPDH, 1 : 2000) and mouse monoclonal anti‐β‐actin (Abcam, ab8226, 1 : 3000) with rabbit polyclonal anti‐mouse Ig‐HRP (Dako antibodies, #P0260, 1 : 3000) were used to control protein loading.

### CSR to IgG1, tail fibroblasts, telomere FISH (T‐FISH), statistical analyses

CSR to IgG1 was performed as previously described in Refs 4, 8, 24. Briefly, splenic B cells were isolated by negative selection using magnetic immunolabelling with an EasySep™ Mouse B Cell Isolation Kit (Stemcell, Cambridge, UK, #19854), stimulated with IL‐4 and lipopolysaccharide (LPS), and analyzed by flow cytometry at day 4. Antibody used for IgG1 detection was anti‐IgG1‐APC (BD Biosciences, Franklin Lakes, NJ, USA, #550874). Primary murine tail fibroblasts were generated and cultured as previously described 4, 5, 7. T‐FISH was performed as previously described 4, 5, 7. Statistical analyses were performed using graphpad prism 7.03 (La Jolla, CA, USA), one‐way ANOVA.

### Histological brain analysis

We isolated brains of 5‐week‐old mice, fixed them with 4% formaldehyde for 15 days, paraffin‐embedded, and sectioned (4 μm). Nissl staining was performed as described in Ref. 25. Briefly, after deparaffinization and hydration, the sections were immersed in 0.1% cresyl violet acetate (Sigma) for 5 min. Then, the samples were rinsed with distilled water and dehydrated in ethanol, cleared in Clear‐Rite™ 3 (Thermo Fisher Scientific, Waltham, MA, USA), and mounted with Entall^®^New (Merck Millipore, Burlington, MA, USA). Images were taken with Nikon D5‐Fi2 microscope (Nikon, Tokyo, Japan). Figures were analyzed to distinguish between apoptotic cells harboring pyknotic nuclei (strong staining, rounded shape, and smaller size) from proliferative cells (strong staining, flattened cells with a lot of cytoplasm around nuclei). Terminal deoxynucleotide transferase‐mediated dUTP nick end labeling (TUNEL) assay was performed by In Situ Cell Death Detection Kit, TMR red Protocol (Roche, Basel, Switzerland) following the manufacturer's guide. Briefly, the sections were deparaffinized by heating and hydrated with decreasing concentrations of ethanol. Then, the samples were treated with proteinase K for 20 min at 37 °C and washed with 1× PBS (Sigma). Sections were incubated in a terminal deoxynucleotidyl transferase (TdT) reaction mix for 1 h at 37 °C, washed with PBS, and mounted with Vectashield (Vector Laboratories, Burlingame, CA, USA). Images were taken with Zeiss LSM 510 Meta microscope (Nikon).

## Results

### Generation of *Paxx* ^*−/−*^ mice

**Figure 1:**
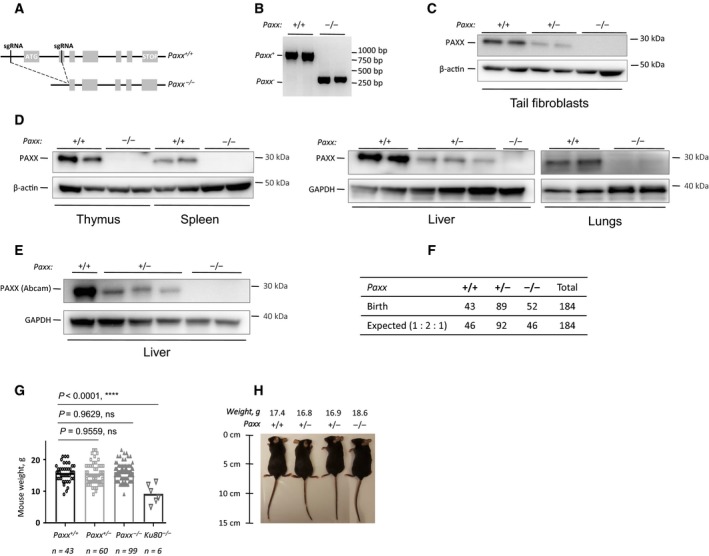
Generation of *Paxx* ^*−/−*^ mice. (A) Top. Schematic diagram of murine *Paxx* locus indicating the ATG and STOP sites, and the targeted loci in the promoter region for sgRNA#1 and in exon 2 for sgRNA#2. Bottom. Resulting *Paxx* ^*−/−*^ locus lacking part of the promoter region, transcription start site, and exon 1. (B) PCR‐based genotyping strategy reveals the *Paxx* wt allele (965 bp) and *Paxx* null allele (270 bp). The following samples are presented, from left to right: two *Paxx* ^*+/+*^ and two *Paxx* ^*−/−*^. (C,D) Western blot analyses using *Novus* anti‐PAXX immunoglobulins revealed the lack of PAXX protein in *Paxx* ^*−/−*^ tail fibroblasts, spleen, thymus, liver, and lungs when compared to *Paxx* ^*+/+*^ controls and reduction of PAXX protein in *Paxx* ^*+/−*^ cells. (E) Western blot using *Abcam* anti‐PAXX immunoglobulins showed no PAXX protein in *Paxx* ^*−/−*^ and reduction of PAXX protein in *Paxx* ^*+/−*^ livers compared to *Paxx* ^*+/+*^. (F) Analyses of 184 pups born from *Paxx* ^*+/−*^ parents revealed expected genetic distribution of *Paxx* ^*+/+*^ (43), *Paxx* ^*+/−*^ (89), and *Paxx* ^*−/−*^ (52) mice, which is close to 1 : 2 : 1 distribution. (G) Body weight of 30‐day‐old *Paxx* ^*+/+*^ mice (*n *=* *43) is not distinguishable from *Paxx* ^*+/−*^ (*n *=* *60, *P *=* *0.9559) and *Paxx* ^*−/−*^ mice (*n *=* *99, *P *=* *0.9629). Six *Ku80* ^*−/−*^ mice of the same age were significantly smaller than *Paxx* ^*+/+*^, *Paxx* ^*+/−*^, and *Paxx* ^*−/−*^ mice, *P *<* *0.0001. (H) Example of 5‐week‐old mice, *Paxx* ^*+/+*^, two *Paxx* ^*+/−*^, and *Paxx* ^*−/−*^, as indicated.

To identify the physiological role of PAXX, we generated a mouse model with deletion of part of the *Paxx* locus on a C57BL6/N background (Fig. 1). For this, the purified sgRNAs and Cas9 RNA were microinjected into the fertilized oocytes, resulting in a locus deletion and complete inactivation of the *Paxx* gene function. A total of 158 injected embryos were reimplanted into foster mothers leading to 60 viable pups. Four *Paxx* F0 null founders that carried 538‐ to 670‐bp deletions covering the *Paxx* promoter and exons 1–2 were identified by PCR screening (Fig. 1B) and DNA sequencing. The founders were backcrossed to C57BL6/N WT mice, and the first heterozygous generation was used to establish colonies. The resulting *Paxx*‐deficient mice (*Paxx* ^*−/−*^) showed complete absence of the PAXX protein in tail fibroblasts, spleen, thymus, liver, and lungs when compared to *Paxx* ^*+/+*^ and *Paxx* ^*+/−*^ littermates (Fig. 1C–E). These results we verified using two independent antibodies, one from NovusBio and one from Abcam, both generated to recognize the C‐terminal half, 109–204 amino acids of human PAXX protein. Moreover, haploinsufficiency for *Paxx* resulted in reduced PAXX protein level in *Paxx* ^*+/−*^ cells compared to *Paxx* ^*+/+*^ controls, as shown for tail fibroblasts and liver (Fig. 1C–E). We concluded that our *Paxx* ^*−/−*^ mice do not express PAXX.

### *Paxx* ^*−/−*^ mice are viable and grow normally

The inactivation of one or both *Paxx* alleles resulted in viable and fertile mice indistinguishable by size from WT littermates (Fig. 1F–H). The pups from *Paxx* ^*+/−*^ parents were born at the expected 1 : 2 : 1 proportion, 43 *Paxx* ^*+/+*^, 89 *Paxx* ^*+/−*^, and 52 *Paxx* ^*−/*−^ mice (Fig. 1F). At day 30, the average size of *Paxx* ^*+/+*^, *Paxx* ^*+/−*^, and *Paxx* ^*−/−*^ mice was not significantly different; all these mice were larger than *Ku80* ^*−/−*^ mice which are known to have reduced body size (Fig. 1G,H). To further describe our *Paxx* ^*−/−*^ mouse model, we performed Nissl staining and TUNEL assay on brain sagittal sections of 5‐week‐old mice. Nissl staining revealed that the morphology of the *Paxx* ^*−/−*^ brain was identical to WT littermates. According to this, the presence of apoptotic cell WT brains did not outnumber the apoptotic cells found in *Paxx* ^*−/−*^. Proliferative cells were found in both genotypes at similar proportion (not shown). TUNEL assay revealed that the levels of apoptosis were almost undetectable in both *Paxx* ^*−/−*^ and WT brains. We concluded that inactivation of *Paxx* alone did not affect growth, size, fertility, and development of central nervous system in mice, which is in line with recently published three independent *Paxx*‐knockout mouse models 17, 18, 19.

### *Paxx* ^*−/−*^ mice develop normal lymphoid organs

**Figure 2:**
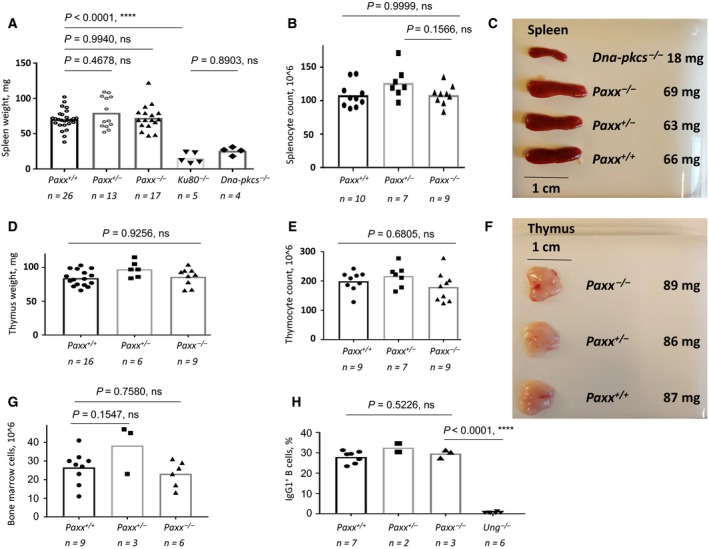
Development of lymphoid organs in *Paxx* ^*−/−*^ mice. (A) The weight of spleens isolated from *Paxx* ^*+/+*^ (*n *=* *26), *Paxx* ^*+/−*^ (*n *=* *13), and *Paxx* ^*−/−*^ (*n *=* *17). Six‐week‐old mice were not significantly different, with *P *>* *0.4677. Spleen size in immunodeficient *Ku80* ^*−/−*^ and *Dna‐pkcs* ^*−/−*^ mice was significantly reduced, correspondingly; both pairs, *Paxx* ^*−/−*^ vs *Ku80* ^*−/−*^, and *Paxx* ^*−/−*^ vs *Dna‐pkcs* ^*−/−*^, have *P *<* *0.0001. (B) Splenocyte count is not affected in *Paxx* ^*−/−*^ mice when compared to WT littermates, *P *>* *0.1566. (C) Example of spleens isolated from *Dna‐pkcs* ^*−/−*^, *Paxx* ^*−/−*^, *Paxx* ^*+/−*^, and *Paxx* ^*+/+*^ mice. (D) The weight of thymus from *Paxx* ^*+/+*^ (*n *=* *16), *Paxx* ^*+/−*^ (*n *=* *6), and *Paxx* ^*−/−*^ (*n *=* *9) is similar with *P *>* *0.9255. (E) Thymocyte count was nearly identical in *Paxx* ^*+/+*^ (*n *=* *9), *Paxx* ^*+/−*^ (*n *=* *7), and *Paxx* ^*−/−*^ (*n *=* *9) mice, *P *>* *0.2649. (F) Example of thymi from *Paxx* ^*−/−*^, *Paxx* ^*+/−*^, and *Paxx* ^*+/+*^ mice. (G) Count of total cells in bone marrow was similar in *Paxx* ^*+/+*^ (*n *=* *9), *Paxx* ^*+/−*^ (*n *=* *3), and *Paxx* ^*−/−*^ (*n *=* *6) mice, *P *>* *0.1546. (H) CSR to IgG1 was identical in *Paxx* ^*+/+*^ (*n *=* *7), *Paxx* ^*+/−*^ (*n *=* *2), and *Paxx* ^*−/−*^ (*n *=* *3) mice. CSR to IgG1 was significantly reduced in *Ung* ^*−/−*^ B cells when compared to *Paxx* ^*−/−*^ (*n *=* *6), *P *<* *0.0001.

Neither spleen nor thymus development was affected in the absence of PAXX (Fig. 2). The average spleen sizes at 45 days were 70, 67, and 73 mg for *Paxx* ^*+/+*^, *Paxx* ^*+/−*^, and *Paxx* ^*−/−*^, respectively (no significant difference), while it was reduced in NHEJ‐deficient *Ku80* ^*−/−*^ (15 mg) and *Dna‐pkcs* ^*−/−*^ (21 mg) mice of the same age, *P *<* *0.0001 (Fig. 2A,C). The average splenocyte counts were similar in *Paxx* ^*+/+*^, *Paxx* ^*+/−*^, and *Paxx* ^*−/−*^ mice (108, 126, and 108 million, respectively, *P *>* *0.1415, Fig. 2B). Thymus weight was also comparable between *Paxx* ^*+/+*^, *Paxx* ^*+/−*^, and *Paxx* ^*−/−*^ mice (84, 101, and 83 mg, respectively) (Fig. 2D,F). The thymocyte count was also similar between *Paxx* ^*+/+*^, *Paxx* ^*+/−*^, and *Paxx* ^*−/−*^ mice (199, 226, 179 million, respectively) (Fig. 2E). Total count of cells in bone marrow was similar in *Paxx* ^*+/+*^, *Paxx* ^*+/−*^, and *Paxx* ^*−/−*^ mice (*P *> 0.1546, Fig. 2G). Thus, we concluded that PAXX is dispensable for development of lymphoid tissue in mice.

### *Ex vivo* stimulated primary *Paxx* ^*−/−*^ B lymphocytes exhibit normal CSR levels

Deleting of XLF and XRCC4 results in a twofold to threefold reduction in CSR activity, which can be explained by the activity of alternative end joining (A‐EJ) in the *Xrcc4* ^*−/−*^ cells 24 and the residual activity of both classical NHEJ and A‐EJ in *Xlf* ^*−/−*^ cells 3, 8. To determine the role of PAXX in CSR, we isolated primary splenic B lymphocytes from *Paxx* ^*+/+*^, *Paxx* ^*+/−*^, and *Paxx* ^*−/−*^ mice and stimulated them with bacterial LPS and interleukin 4 to undergo CSR. Four days after stimulation, CSR levels to IgG1 in *Paxx* ^*+/+*^ and *Paxx* ^*−/−*^ cells ranged from 23.4% to 31.3% and were not significantly different (Fig. 2H), *P *=* *0.5226. B cells from *Ung* ^*−/−*^ mice were used as negative control and switched at background levels, 0.4–1.2% of IgG1^+^ B cells, in line with original observations 26 (Fig. 2H). We therefore concluded that PAXX is dispensable for CSR in mice.

### *Paxx* ^*−/−*^ mice exhibit no change in genomic stability

**Figure 3:**
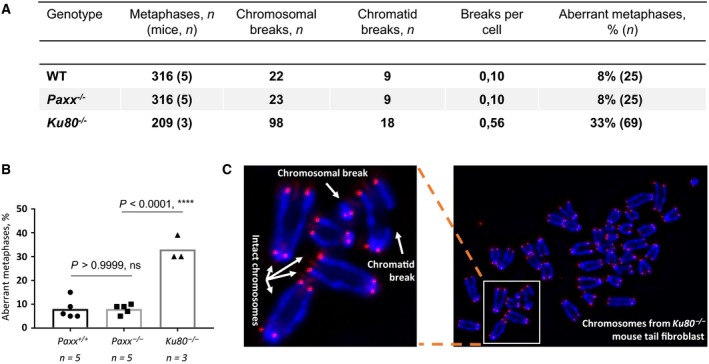
Analyses of genomic instability in *Paxx* ^*−/−*^ tail fibroblasts. (A) Summary of genomic instability in *Paxx* ^*+/+*^ (WT,* n *=* *5), *Paxx* ^*−/−*^ (*n *=* *5), and *Ku80* ^*−/−*^ (*n *=* *3) mouse tail fibroblasts. (B) Proportion of aberrant metaphases in tail fibroblasts of indicated genotypes. (C) Example of metaphase spread from one *Ku80* ^*−/−*^ mouse tail fibroblast (right). Intact chromosomes with four telomeric signals, chromosomal and chromatid breaks with 2, 3, and 1 telomeric signals, correspondingly, are indicated at the selected area (left).

We have previously demonstrated that inactivation of the core NHEJ factor *Ku70* results in a threefold to sixfold increase of aberrant metaphases in murine tail fibroblasts measured as chromosomal and chromatid breaks compared to WT controls. In addition, inactivation of *Xlf* and *Dna‐pkcs* resulted in a significant though moderate increase in the proportion of aberrant metaphases 1, 4, 5, 7. To determine whether *Paxx* inactivation affects genomic stability, we measured metaphase aberrations in isolated tail fibroblasts from five *Paxx* ^*+/+*^ and five *Paxx* ^*−/−*^ mice, using three *Ku80* ^*−/−*^ mice as NHEJ‐deficient controls. We found that the average proportion of aberrant metaphases was identical in *Paxx* ^*+/+*^ and *Paxx* ^*−/−*^ mice (8%, *P *>* *0.9999), while significantly increased to 33% in *Ku80* ^*−/−*^ mice (*P *<* *0.0001) (Fig. 3). We concluded that PAXX is dispensable for genomic stability in mice.

## Discussion

Here, we report the newly generated *Paxx* ^*−/−*^ knockout mouse model. We deleted part of the *Paxx* promoter region, transcription start site, and exons1–2. We did not delete the entire *Paxx* locus because it overlaps with other genes. In particular, prostaglandin‐H2 D‐isomerase (*Ptgds*) is in sense orientation 5.6 kb upstream of *Paxx*, chloride intracellular channel protein 3 (*Clic3*) overlaps with *Paxx* intron 3, and ATP‐binding cassette subfamily A member 2 (*Abca2*) is in antisense orientation 6.6 kb downstream of *Paxx*.

Inactivation of both alleles of *Paxx* in mice resulted in no detectable protein levels in tail fibroblasts, spleen, thymus, liver, and lungs. Furthermore, it did not affect growth, fertility, development of lymphoid organs, or genomic stability of mice, when compared to heterozygous or WT littermates. In addition, PAXX was dispensable for CSR to IgG1 in primary B splenocytes, which is in line with data obtained using knockout B‐cell CH12F3 cell lines, where inactivation of *Paxx* did not affect CSR to IgA [14,27]. Finally, we analyzed brain sections of WT and *Paxx* ^*−/−*^ mice and found no difference in neurodevelopment using two independent methods, Nissl staining and TUNEL assay (not shown).

While our mouse model was produced and analyzed, three other groups independently reported *Paxx*‐deficient mice 17, 18, 19. In contrast to our mouse model, those mice had the entire *Paxx* locus deleted. These groups found that PAXX is dispensable for mouse development unless its paralogue XLF is also inactivated. Moreover, *Paxx* ^*−/−*^ *Xlf* ^*−/−*^ mice are embryonically lethal, which limits further studies of the functional interaction between PAXX and XLF in adult animals. One option to bypass this challenge is to generate conditional knockout genes for *Xlf, Paxx*, or both genes. This would enable the specific deletion of *Xlf a*nd *Paxx* in organs of interest, such as the spleen, thymus, or brain. However, conditional knockouts are often limited by, for example, incomplete deletion. Previously, it has been demonstrated that the inactivation of *Ku80* rescues embryonic lethality in *Lig4* ^*−/−*^ mice 28, and *Ku70* inactivation rescues perinatal lethality in *Xlf* ^*−/−*^ *Dna‐pkcs* ^*−/−*^ 5. Thus, the inactivation of upstream NHEJ factors Ku70 or Ku80 might rescue embryonic lethality in *Paxx* ^*−/−*^ *Xlf* ^*−/−*^ mice, although it would completely inactivate residual classical NHEJ. Additionally, haploinsufficiency for the *Trp53* gene (p53) rescues embryonic lethality of *Lig4* ^*−/−*^ 29, *Xrcc4* ^*−/−*^ 30, and *Xlf* ^*−/−*^ *Dna‐pkcs* ^*−/−*^ mice 5. Thus, we may speculate whether a *Paxx* ^*−/−*^ *Xlf* ^*−/−*^ *Trp53* ^*+/−*^ mouse may be viable despite complete absence of PAXX and XLF in all organs and tissues allowing the study of functional interaction between *Paxx* and *Xlf in vivo*.

## Author contributions

All authors designed research, analyzed results, performed experiments, and commented on the manuscript. VO wrote the manuscript.

## References

[1] Kumar V , Alt FW and Oksenych V (2014) Functional overlaps between XLF and the ATM‐dependent DNA double strand break response. DNA Repair (Amst) 16, 11–22. 24674624 10.1016/j.dnarep.2014.01.010 PMC4017585

[2] Alt FW , Zhang Y , Meng FL , Guo C and Schwer B (2013) Mechanisms of programmed DNA lesions and genomic instability in the immune system. Cell 152, 417–429. 23374339 10.1016/j.cell.2013.01.007 PMC4382911

[3] Li G , Alt FW , Cheng HL , Brush JW , Goff PH , Murphy MM , Franco S , Zhang Y and Zha S (2008) Lymphocyte‐specific compensation for XLF/cernunnos end‐joining functions in V(D)J recombination. Mol Cell 31, 631–640. 18775323 10.1016/j.molcel.2008.07.017 PMC2630261

[4] Oksenych V , Kumar V , Liu X , Guo C , Schwer B , Zha S and Alt FW (2013) Functional redundancy between the XLF and DNA‐PKcs DNA repair factors in V(D)J recombination and nonhomologous DNA end joining. Proc Natl Acad Sci U S A 110, 2234–2239. 23345432 10.1073/pnas.1222573110 PMC3568359

[5] Xing M , Bjørås M , Daniel JA , Alt FW and Oksenych V (2017) Synthetic lethality between murine DNA repair factors XLF and DNA‐PKcs is rescued by inactivation of Ku70. DNA Repair (Amst) 57, 133–138. 28759779 10.1016/j.dnarep.2017.07.008 PMC5584571

[6] Liu X , Jiang W , Dubois RL , Yamamoto K , Wolner Z and Zha S (2012) Overlapping functions between XLF repair protein and 53BP1 DNA damage response factor in end joining and lymphocyte development. Proc Natl Acad Sci USA 109, 3903–3908. 22355127 10.1073/pnas.1120160109 PMC3309750

[7] Oksenych V , Alt FW , Kumar V , Schwer B , Wesemann DR , Hansen E , Patel H , Su A and Guo C (2012) Functional redundancy between repair factor XLF and damage response mediator 53BP1 in V(D)J recombination and DNA repair. Proc Natl Acad Sci U S A 109, 2455–2460. 22308489 10.1073/pnas.1121458109 PMC3289340

[8] Zha S , Guo C , Boboila C , Oksenych V , Cheng HL , Zhang Y , Wesemann DR , Yuen G , Patel H , Goff PH *et al* (2011) ATM damage response and XLF repair factor are functionally redundant in joining DNA breaks. Nature 469, 250–254. 21160472 10.1038/nature09604 PMC3058373

[9] Lescale C , Abramowski V , Bedora‐Faure M , Murigneux V , Vera G , Roth DB , Revy P , De Villartay JP and Deriano L (2016) RAG2 and XLF/Cernunnos interplay reveals a novel role for the RAG complex in DNA repair. Nat Commun 7, 10529. 26833222 10.1038/ncomms10529 PMC4740868

[10] Craxton A , Somers J , Munnur D , Jukes‐Jones R , Cain K and Malewicz M (2015) XLS (c9orf142) is a new component of mammalian DNA double‐stranded break repair. Cell Death Differ 22, 890–897. 25941166 10.1038/cdd.2015.22 PMC4423191

[11] Ochi T , Blackford AN , Coates J , Jhujh S , Mehmood S , Tamura N , Travers J , Wu Q , Draviam VM , Robinson CV *et al* (2015) DNA repair. PAXX, a paralog of XRCC4 and XLF, interacts with Ku to promote DNA double‐strand break repair. Science 347, 185–188. 25574025 10.1126/science.1261971 PMC4338599

[12] Xing M , Yang M , Huo W , Feng F , Wei L , Jiang W , Ning S , Yan Z , Li W , Wang Q *et al* (2015) Interactome analysis identifies a new paralogue of XRCC4 in non‐homologous end joining DNA repair pathway. Nat Commun 6, 6233. 25670504 10.1038/ncomms7233 PMC4339890

[13] Hung PJ , Chen BR , George R , Liberman C , Morales AJ , Colon‐Ortiz P , Tyler JK , Sleckman BP and Bredemeyer AL (2017) Deficiency of XLF and PAXX prevents DNA double‐strand break repair by non‐homologous end joining in lymphocytes. Cell Cycle 16, 286–295. 27830975 10.1080/15384101.2016.1253640 PMC5323033

[14] Kumar V , Alt FW and Frock RL (2016) PAXX and XLF DNA repair factors are functionally redundant in joining DNA breaks in a G1‐arrested progenitor B‐cell line. Proc Natl Acad Sci U S A 113, 10619–10624. 27601633 10.1073/pnas.1611882113 PMC5035843

[15] Lescale C , Hasse HL , Blackford AN , Balmus G , Bianchi JJ , Yu W , Bacoccina L , Jarade A , Clouin C , Sivapalan R *et al* (2016) Specific roles of XRCC4 paralogs PAXX and XLF during V(D)J recombination. Cell Rep 16, 2967–2979. 27601299 10.1016/j.celrep.2016.08.069 PMC5033762

[16] Tadi SK , Tellier‐Lebègue C , Nemoz C , Drevet P , Audebert S , Roy S , Meek K , Charbonnier JB and Modesti M (2016) PAXX is an accessory c‐NHEJ factor that associates with Ku70 and Has overlapping functions with XLF. Cell Rep 17, 541–555. 27705800 10.1016/j.celrep.2016.09.026

[17] Abramowski V , Etienne O , Elsaid R , Yang J , Berland A , Kermasson L , Roch B , Musilli S , Moussu JP , Lipson‐Ruffert K *et al* (2018) PAXX and Xlf interplay revealed by impaired CNS development and immunodeficiency of double KO mice. Cell Death Differ 25, 444–452. 29077092 10.1038/cdd.2017.184 PMC5762856

[18] Balmus G , Barros AC , Wijnhoven PW , Lescale C , Hasse HL , Boroviak K , Le Sage C , Doe B , Speak AO , Galli A *et al* (2016) Synthetic lethality between PAXX and XLF in mammalian development. Genes Dev 30, 2152–2157. 27798842 10.1101/gad.290510.116 PMC5088564

[19] Liu X , Shao Z , Jiang W , Lee BJ and Zha S (2017) PAXX promotes KU accumulation at DNA breaks and is essential for end‐joining in XLF‐deficient mice. Nat Commun 8, 13816. 28051062 10.1038/ncomms13816 PMC5216128

[20] Nussenzweig A , Chen C , da Costa Soares V , Sanchez M , Sokol K , Nussenzweig MC and Li GC (1996) Requirement for Ku80 in growth and immunoglobulin V(D)J recombination. Nature 382, 551–555. 8700231 10.1038/382551a0

[21] Gao Y , Chaudhuri J , Zhu C , Davidson L , Weaver DT and Alt FW (1998) A targeted DNA‐PKcs‐null mutation reveals DNA‐PK‐independent functions for KU in V(D)J recombination. Immunity 9, 367–376. 9768756 10.1016/s1074-7613(00)80619-6

[22] Bosma GC , Custer RP and Bosma MJ (1983) A severe combined immunodeficiency mutation in the mouse. Nature 301, 527–530. 6823332 10.1038/301527a0

[23] Nilsen H , Rosewell I , Robins P , Skjelbred CF , Andersen S , Slupphaug G , Daly G , Krokan HE , Lindahl T and Barnes DE (2000) Uracil‐DNA glycosylase (UNG)‐deficient mice reveal a primary role of the enzyme during DNA replication. Mol Cell 5, 1059–1065. 10912000 10.1016/s1097-2765(00)80271-3

[24] Boboila C , Oksenych V , Gostissa M , Wang JH , Zha S , Zhang Y , Chai H , Lee CS , Jankovic M , Saez LM *et al* (2012) Robust chromosomal DNA repair via alternative end‐joining in the absence of X‐ray repair cross‐complementing protein 1 (XRCC1). Proc Natl Acad Sci U S A 109, 2473–2478. 22308491 10.1073/pnas.1121470109 PMC3289296

[25] Liu J , Zhao Y , Yang J , Zhang X , Zhang W and Wang P (2017) Neonatal repeated exposure to isoflurane not sevoflurane in mice reversibly impaired spatial cognition at Juvenile‐Age. Neurochem Res 42, 595–605. 27882447 10.1007/s11064-016-2114-7

[26] Rada C , Williams GT , Nilsen H , Barnes DE , Lindahl T and Neuberger MS (2002) Immunoglobulin isotype switching is inhibited and somatic hypermutation perturbed in UNG‐deficient mice. Curr Biol 12, 1748–1755. 12401169 10.1016/s0960-9822(02)01215-0

[27] Dewan A , Xing M , Lundbæk MB , Gago‐Fuentes R , Beck C , Aas PA , Liabakk N‐B , Sæterstad S , Chau KTP , Kavli BM *et al* (2018) Robust DNA repair in PAXX‐deficient mammalian cells. FEBS Open Bio, https://doi.org/10.1002/2211-5463.12380 10.1002/2211-5463.12380 PMC5832976 29511621

[28] Karanjawala ZE , Adachi N , Irvine RA , Oh EK , Shibata D , Schwarz K , Hsieh CL and Lieber MR (2002) The embryonic lethality in DNA ligase IV‐deficient mice is rescued by deletion of Ku: implications for unifying the heterogeneous phenotypes of NHEJ mutants. DNA Repair (Amst) 1, 1017–1026. 12531011 10.1016/s1568-7864(02)00151-9

[29] Frank KM , Sharpless NE , Gao Y , Sekiguchi JM , Ferguson DO , Zhu C , Manis JP , Horner J , DePinho RA and Alt FW (2000) DNA ligase IV deficiency in mice leads to defective neurogenesis and embryonic lethality via the p53 pathway. Mol Cell 5, 993–1002. 10911993 10.1016/s1097-2765(00)80264-6

[30] Gao Y , Ferguson DO , Xie W , Manis JP , Sekiguchi J , Frank KM , Chaudhuri J , Horner J , DePinho RA and Alt FW (2000) Interplay of p53 and DNA‐repair protein XRCC4 in tumorigenesis, genomic stability and development. Nature 404, 897–900. 10786799 10.1038/35009138

